# Constitutive AKT activation in follicular lymphoma

**DOI:** 10.1186/1471-2407-14-565

**Published:** 2014-08-05

**Authors:** Ouardia I Yahiaoui, Jacques A Nunès, Céline Castanier, Raynier Devillier, Florence Broussais, Aurélie J Fabre, Dalila Naimi, Réda Bouabdallah, Daniel Olive, Luc Xerri

**Affiliations:** Inserm, U1068, Centre de Recherche en Cancérologie de Marseille, Marseille, France; Institut Paoli-Calmettes, Marseille, France; CNRS, UMR7258, Centre de Recherche en Cancérologie de Marseille, Marseille, France; Aix-Marseille Université, Marseille, France; Université Constantine 1, Laboratoire de génie microbiologique et applications, équipe de biologie physiologie cellulaire et moléculaire, Constantine, Algeria; Department of Hematology, Institut Paoli-Calmettes, Marseille, France; Department of Biopathology, Institut Paoli-Calmettes, Marseille, France; Inserm UMR S910, Department of Medical Genetics and Functional Genomics, Faculté de la Timone, 27 Bd Jean Moulin, 13005 Marseille, France; Centre de Recherche en Cancérologie de Marseille, 27 Bd Leï Roure -BP 30059, 13273 Marseille cedex 9, France

**Keywords:** Follicular lymphoma, PIK3CA mutations, AKT phosphorylation, PTEN

## Abstract

**Background:**

The phosphoinositide 3- kinase (PI3K) pathway is involved in the growth of various human cancers, including lymphoid malignancies. However its role in the pathogenesis of follicular lymphoma (FL) has not been yet described.

**Methods:**

To clarify this point, biopsy tissue samples from 38 human FL cases were investigated for PIK3CA somatic mutations in exon 9 and 20 using direct sequencing. The same samples were analyzed using western blotting and immunohistochemistry to detect expression of AKT, phosphorylated AKT (pAKT), and PTEN proteins. Two cases of benign lymphadenitis were used as controls.

**Results:**

AKT expression was present in all FL and lymphadenitis cases. 14/38 (37%) FL and 2/2 lymphadenitis cases expressed pAKT. 9/38 (24%) FL samples showed high level of pAKT, whereas 5/38 (13%) FL cases and 2/2 benign lymphadenitis samples expressed low level of pAKT. PTEN expression was observed in 30/38 (79%) FL and 2/2 benign lymphadenitis cases, whereas 8/38 (21%) FL cases showed loss of PTEN expression. 3 cases with positive pAKT did not express PTEN. PIK3CA mutations were not detected in any sample.

**Conclusions:**

These data suggest that the PI3K/AKT signaling pathway could be activated in a subset of FL cases, due to either AKT phosphorylation or PTEN downregulation, in the absence of PIK3CA mutations.

## Background

Follicular lymphoma is the most frequent occurring form of low grade of Non Hodgkin lymphoma (NHL) and account for approximately 20% of NHL cases [1]. The clinical course of FL is relatively indolent when chemotherapeutic agents are combined with rituximab [2]. Nonetheless, a significant proportion of cases either relapses or transform into aggressive diffuse large B cell lymphomas (DLBCL) [2]. Thus there is a need for more efficient therapies to improve the outcome of FL patients. The use of immunomodulating agents has recently proved to be of clinical interest [3]. Other targets related to FL pathogenesis could offer new opportunities.

FL is derived from germinal center B cells and characterized in most cases by the chromosomal translocation t(14; 18) (q32; q21), causing deregulated expression of the anti-apoptotic Bcl2 protein [1]. This translocation is considered as an initiating event in the molecular pathogenesis in the FL, but is not sufficient in the development of FL [1]. Additional pathogenic events that are required for the manifestation of FL remain poorly understood. They may be related to molecular mechanisms involved in the regulation of physiological process including cell proliferation, survival, angiogenesis and tumor growth, such as the PI3K/AKT/mTOR pathway [4, 5].

PI3Ks are a family of lipid kinases classified into three major subfamilies. The PI3K class I is activated by cell surface receptors and consists of two subfamilies, class IA and class IB, which are composed of heterodimers of catalytic and regulatory subunits, identified as p110(α, β, δ)/p85(α, β) and p110γ/p101 for class IA and class IB, respectively [6, 7]. Class II PI3Ks are monomeric isoforms p110-like catalytic subunit that can be activated by RTK, cytokine receptors, and integrin. The class III includes heterodimeric enzymes composed of VPs34 catalytic and p150 adaptor PI3K subunits [6, 7].

In response to growth factors, protein tyrosine kinases receptors can recruit and activate PI3K, which in turn induces an increase in phosphatidylinositol-3, 4, 5-trisphosphate (PIP3) levels. The phosphatase and tensin homolog (PTEN) protein dephosphorylates PIP3 to PIP2, acting as an antagonist of PI3K. PIP3 transduces intracellular signaling by recruiting and participating to the phosphorylation of variety of proteins including the serine/threonine kinase AKT. Subsequently, activated AKT may phosphorylate a range of substrates, thereby activating these targets and favoring cell survival [8, 9].

Constitutive activation of the PI3K/AKT pathway occurs in various human cancers due to genetic aberrations. They include mutation or amplification of the catalytic subunit p110α encoded by PIK3CA gene [10–15], loss of PTEN function through mutations, deletions, promoter methylation silencing, or protein instability [16, 17]. Similarly, gain of function of AKT can occur by amplification, overexpression, and increased phosphorylation [18–20], or mutation of p85α regulatory subunit of PI3K [21, 22]. Activating mutations of PIK3CA p110α are among the most frequent alterations in human cancers [23, 24].

Only a few studies have reported dysregulation of the PI3K/AKT pathway in lymphoid malignancies. PIK3CA mutations and PTEN inactivation were detected in DLBCLs, and high pAKT expression was associated with poor survival [25, 26]. Mantle cell lymphomas (MCL) were shown to lack PIK3CA mutations, but often display constitutive AKT activation, resulting from loss of PTEN expression in some cases [27]. Loss of PTEN expression and/or PIK3CA gene amplification were found to be mutually exclusive mechanisms of AKT activation in the pathogenesis of MCL [28].

FL tissue samples analyzed using proteomic analysis showed increased expression of phosphorylated AKT at the position Ser473 [29, 30]. Besides, there is a relative effectiveness of PI3K and mTOR inhibitors (NVP-BEZ235) on human lymphoma cell lines [2].

Recently, idelalisib (CAL- 101), a PI3Kδ inhibitor was shown to downregulate pAKT expression and to induce apoptosis in FL cell lines [31], leading to a phase I study with promising results in patients with Non Hodgkin Lymphoma [32] that will be extended in phase II study [33]. Altogether these previous works indicate that dysfunction of PI3K/AKT pathway might be involved in the pathogenesis of FL by some mechanisms that remain unclear to date.

In the present study, we have sought to better characterize this putative PI3K/AKT dysfunction in a series of FL tissue samples using a combination of methods including DNA sequencing, western blot (WB) and immunohistochemistry (IHC).

## Methods

### Tumor tissue samples and patients

Frozen tissue samples were collected between 2002 and 2012 from 38 FL patients at the time of diagnosis, prior to any treatment. Immunophenotyping at the time of diagnosis showed that all FL cases contained more than 70% of tumor B cells (based on immunohistochemical CD20 expression), whereas the reactive T-cell component identified by CD3 positivity was lower than 30%. Two benign lymphadenitis samples were used as controls. All patients gave informed consent and the study was approved by the ethical board of the Paoli-Calmettes institute.

The corresponding formalin fixed and paraffin embedded samples were also available. The diagnosis of FL was made according to the World Health Organization classification [34], the FL samples were classified as grade 1-2 (n = 34), grade 3 (n = 2) and not gradable (n = 2). The clinical characteristics of the patients are detailed in Table 1. Median follow-up of patients was 94 months.

**Table 1:** **Clinical characteristics and pAKT expression status of FL patients**

		Negative pAKT		Positive pAKT		
	Total	n	%	n	%	***P***
**NO, patients**	**36**	**22**	**61**	**14**	**39**	
**Sex**	**Male**	**10**	**45**	**6**	**43**	**0.577**
	**Female**	**12**	**55**	**8**	**57**	
**Age**	**< 60 years**	**15**	**68**	**10**	**71**	**0.569**
	**≥ 60 years**	**7**	**32**	**4**	**29**	
	**Median [range]**	**56 [30-72]**		**52 [34-68]**		**0.575**
**Lactate dehydrogenase**	**N**	**19**	**86**	**8**	**57**	**0.03**
	**>N**	**2**	**9**	**6**	**43**	
	**Unknown**	**1**	**5**	**0**	**0**	
**Performance status**	**0**	**7**	**32**	**2**	**14**	**0.045**
	**1**	**0**	**0**	**3**	**22**	
	**Unknown**	**15**	**68**	**9**	**64**	
**B symptoms**	**0**	**5**	**23**	**4**	**29**	**0.601**
	**1**	**4**	**18**	**4**	**29**	
	**Unknown**	**13**	**59**	**6**	**42**	
**Ann Arabor stage**	**I-II**	**7**	**32**	**2**	**14**	**0.254**
	**III-IV**	**15**	**68**	**11**	**79**	
	**Unknown**	**0**	**0**	**1**	**7**	
**Adenopathy**	**0-3**	**14**	**64**	**7**	**50**	**0.282**
	**4 or more**	**6**	**27**	**6**	**43**	
	**Unknown**	**2**	**9**	**1**	**7**	
**Hemoglobin**	**≥ 12 g/dl**	**13**	**59**	**9**	**64**	**0.684**
	**< 12 g/dl**	**3**	**14**	**2**	**14**	
	**Unknown**	**6**	**27**	**3**	**22**	
**β2 microglobulin**	**N**	**9**	**41**	**5**	**36**	**0.306**
	**> N**	**4**	**18**	**5**	**36**	
	**Unknown**	**9**	**41**	**4**	**28**	
**Bone marrow involvement**	**0**	**9**	**41**	**7**	**50**	**0.58**
	**1**	**10**	**45**	**7**	**50**	
	**Unknown**	**3**	**14**	**0**	**0**	
**FLIPI**	**0-1**	**3**	**14**	**1**	**7**	**0.390**
	**2**	**7**	**32**	**4**	**29**	
	**≥ 3**	**4**	**18**	**6**	**43**	
	**Unknown**	**8**	**36**	**3**	**21**	
**Follow up (months)**	**Median [range]**	**90 [9-200]**		**97 [50-200]**		**0.687**

### DNA, RNA, and protein extraction

Genomic DNA, RNA, and proteins were extracted from frozen tissues using AllPrep DNA/RNA/Protein purification kit (QIAGEN) according to the manufacturer’s protocol. The concentration and purity of DNA and RNA of each sample were measured using a Nanodrop.

### Polymerase Chain Reaction (PCR) and PIK3CA hotspot mutations analysis

The PCR mixture contained 25 ng of DNA, 0.4 μM of each primer (forward and reverse for exon 9 or 20, see below for sequences), 0.2 mM of dNTP, 0.04 U of Taq DNA polymerase, 1.5 mM MgCl_2_ in TE buffer. To amplify exon 20, after an initial activation at 95°C for 10 minutes, 35 cycles were done, which include 30 seconds at 95°C for denaturation step, 30 seconds for annealing step at 55°C and 30 seconds for elongation step at 72°C.

Finally, termination step followed at 72°C for 10 minutes. To amplify exon 9, a touch-down PCR was done to avoid amplification of a non-specific PCR product. Activation, denaturation, elongation and termination steps were the same as exon 20 but annealing temperatures changed: 4 cycles at 60°C, 4 cycles at 59°C, 4 cycles at 58°C, 6 cycles at 57°C, 6 cycles at 56°C, 8 cycles at 55°C and 13 cycles at 54°C. PCR products were then separated by 2% agarose gel electrophoresis to visualize the appropriate bands. The PCR products were cleaned-up using a Multiscreen plate (Millipore) according to the manufacturer’s protocol and subjected to sequencing reaction in forward and reverse directions using ABI Big Dye Terminator V1.1 and an ABI 3730 automated capillary sequencer (Applied Biosystem). The data were analyzed with SeqScape software. The primers used for PCR and sequencing were as follows: exon 9, 5'-TTGCTTTTTCTGTAAATCATCTGT-3' (forward) and 5'-CTGCTTTATTTATTCCAATAGGTATG-3' (reverse); exon 20, 5'-CTCAATGATGCTTGGCTCTG-3' (forward) and 5'-GGAATCCAGAGTGAGCTTTC-3' (reverse).

### Western blot analysis

Protein pellets were isolated after purification of genomic DNA and RNA according to the manufacturer’s instructions, and were then dissolved in 2 × Laemmli buffer. Protein samples were heated at 95°C for 5 minutes and resolved by standard 9% SDS-polyacrylamide gels. Immunoblots were performed as previously described [35]. The primary antibodies used are commercially available, anti- AKT (Cell Signaling Technology (CST), # 9272, used at 1:1800 dilution) anti- phospho Ser473-AKT (CST, # 4058, used at 1:1000 dilution), anti- PTEN (CST, # 9552, clone 138G6, used at 1:1000 dilution) and anti-GAPDH (Abcam, clone mAbcam 9484, used at 1:5000 dilution). These antibodies were used in TBS - 0.1% Tween 20 - 5% BSA, overnight at 4°C under agitation. Lysates from the Jurkat (loss of PTEN) and SupT1 (PTEN-positive) T cell lines were used as positive and negative controls for AKT phosphorylation, and PTEN expression, respectively.

The AKT protein was used as a loading control to evaluate the expression of phosphorylated form of AKT (pAKT) in each sample. The same method was used to evaluate PTEN expression, except that GAPDH was used as an additional loading control.

Densitometric analysis of AKT and pSer473-AKT bands were quantified using ImageJ software and the ratio pSer473-AKT/AKT values of each sample were normalized to the value of Jurkat cell condition as positive control.

### Immunohistochemistry

4 μm paraffin sections were cut, mounted on glass slides and air dried overnight at room temperature. The staining protocol was performed using an Envision FLEX/Dako Autostainer system according to the manufacturer’s supplied instructions (Dako, Glostrup, Denmark). Antigen retrieval was accomplished by heating the slides in EnVision FLEX target retrieval solution pH 6 (Dako) at 99°C for 40 minutes. The slides were cooled down at room temperature for 20 minutes, washed for 5 minutes in the EnVision FLEX wash buffer. Endogenous peroxidase activity and non-specific binding sites were blocked with EnVision FLEX Peroxidase-Blocking Reagent (Dako) for 5 minutes. The slides were then washed with EnVision FLEX wash buffer, then incubated with either the mouse mAb anti-pSer473-AKT (CST, # 4051, clone 587 F11, used at 1:200 dilution), or with the mouse mAb anti-PTEN (Dako, clone 6H2.1, used at 1:150 dilution) at room temperature for 60 minutes, followed by washing in the EnVision FLEX wash buffer.

To obtain an optimal amplification of the PTEN staining, slides were incubated with the EnVision FLEX Mouse LINKER (Dako) for 15 minutes. After washing, immunodetection was achieved using Envision FLEX system [EnVision FLEX HRP for 20 minutes, followed by incubation in the EnVision FLEX DAB + Chromogen solution (3, 3’-diaminobenzidine tetrahydrochloride, Dako) for 10 minutes], rinsed with EnVision FLEX wash buffer to remove residue 3, 3'-diaminobenzidine tetrahydrochloride, counterstained with EnVision FLEX Hematoxylin for 5 minutes. Positive controls were breast tumors expressing pAKT and normal uterus samples expressing PTEN.

### Statistical analysis

The association of pAKT expression with patient’s characteristics was performed using chi-squared (*x*^*2*^) tests for categorical data. Overall survival (OS) was measured from date of diagnosis to date of death or until the date of last follow up visit for alive patients. Progression free survival (PFS) was defined as the response time after the first chemotherapy. Survival analysis was carried out using Kaplan-Meier method and the log rank test was used when comparing groups. Statistical data was produced by the software SPSS.

## Results

### Absence of activating mutation of the PIK3CA gene in FL

Mutational analysis of the somatic mutations within exon 9 and 20 of PIK3CA was done to assess the two hotspots regions in the helical (exon 9) and kinase (exon 20) domains, in which 80% of the reported mutations are found [10]. We did not detect any mutations in our 38 FL cases.

### Expression of AKT, activated AKT, and PTEN in FL

#### Western blot

**Figure 1:**
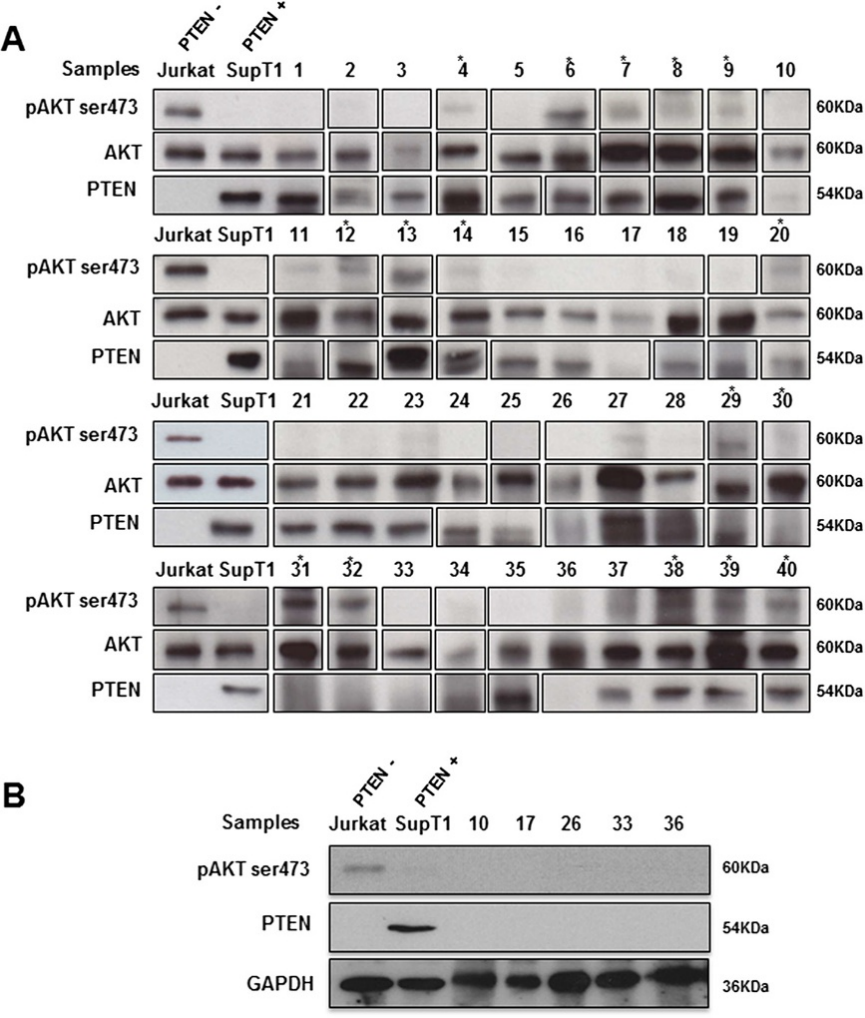
**Representative western blot analysis of AKT, activated AKTSer473, and PTEN in 38 FL samples (#1-38) and lymphadenitis cases (#39 and #40).** AKT was detected in all examined samples, and is shown as a loading control for pAKT detection **(panel A)**. GAPDH is also used as a loading control for PTEN detection **(panel B)**. Asterisks indicate positive samples for pAKT; pAKT expression was detected at high level in samples #6, #7, #9, #13, #14, #29, #31, #32, and #38; and at low level in samples #4, #8, #12, #20, #30, #39, and #40. Jurkat cells served as a positive control for pAKT expression. SupT1 cells served as a positive control for PTEN expression. These results shown are representative of 3 independent experiments.

**Figure 2:**
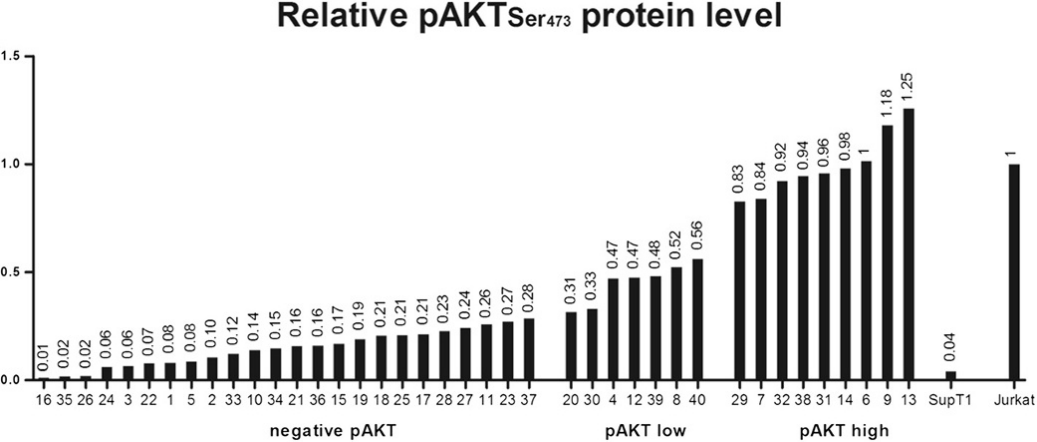
**Quantification of relative p-Ser473 AKT (pAKT) protein amount in FL samples (#1-38) and lymphadenitis cases (#39 and #40).** Samples are ordered according to their p-Ser473AKT level of expression (values are indicated above each bar). The numbering of samples is similar to Figure 1. Jurkat (positive control) and SupT1 (negative control) correspond to the expected profile.

To investigate the activation status of PI3K/AKT pathway in follicular lymphoma, we assessed the expression of AKT and phosphorylated AKT (Ser473), and PTEN protein expression level using WB and IHC. WB analysis revealed in all 38 FL frozen samples a 60 kDa band evidencing AKT expression. Expression of pAKT was observed in 14/38 (37%) FL samples (Figure 1A) at a similar molecular weight of 60 kDa.Densitometric measurement of the relative intensity of pAKT bands categorizes our series into three groups: 9/38 (24%) FL cases with high level of pAKT protein, 5/38 (13%) FL cases with low level of pAKT protein, and 24/38 (63%) FL cases negative for pAKT expression (Figure 2). In the positive control (Jurkat cells) and negative control (SupT1 cells), the relative value of pAKT protein was considered as 1, and 0.04, respectively. It was estimated between 0.83 and 1.25 for the FL group with high pAKT expression level. In comparison the value was evaluated between 0.31 and 0.56 in FLs with low pAKT level, and between 0.01 and 0.28 in the negative pAKT expression group (Figure 2).To further study the mechanisms of AKT activation in FL, we evaluated PTEN profile expression in the same samples. WB analysis showed that 30/38 (79%) FL expressed PTEN at 54 kDa, whereas 8/38 (21%) of cases showed no expression of PTEN (Figure 1A). Of the 8 cases with loss of PTEN, only 3 were associated with pAKT expression, including 2 cases (cases #31 and #32) with high level of AKT activation, and 1 case (case #30) with low level of AKT activation (Figure 1A), whereas 5 of the 8 cases with loss of PTEN had no pAKT expression. Loss of PTEN and the absence of serine phosphorylation of AKT at the position 473 (pAKT) in these 5 cases were further validated in an independent WB analysis, in which GAPDH was used as a loading control (Figure 1B).Eleven out of 14 FL cases with positive pAKT expressed PTEN. WB showed expression of AKT and PTEN proteins in the lymphadenitis samples (cases #39 and #40) (Figure 1A), which also expressed phosphorylated AKT at low level. Relative value of pAKT was estimated as 0.48 and 0.56 for case #39 and case #40, respectively (Figure 2).

#### Immunohistochemistry

**Figure 3:**
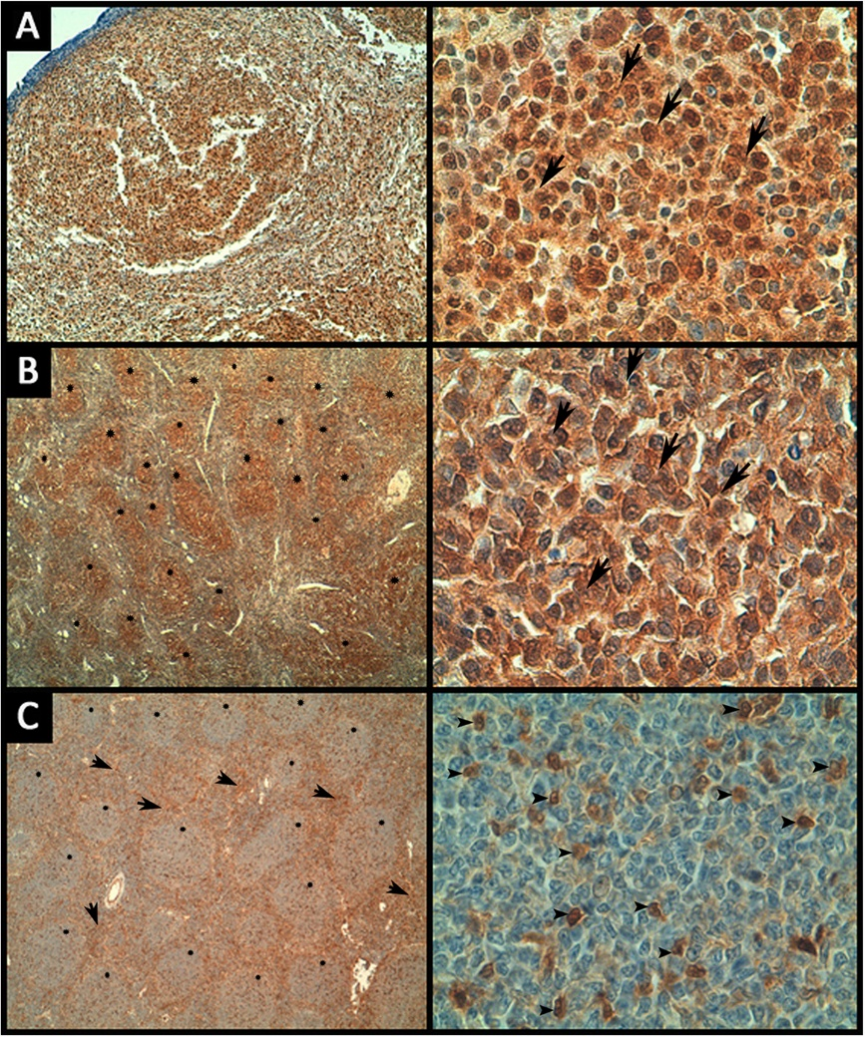
**Representative images of immunohistochemical staining of pAKT and PTEN in FL samples. (A)** Strong nuclear and cytoplasmic pAKT staining in lymphoma cells (arrows) (magnification is × 40 and × 630 for the left and right panels, respectively). **(B)** Strong PTEN staining in lymphoma cells (arrows) within the malignant follicles (stars) (magnification is × 40 and × 630 for the left and right panels, respectively). **(C)** Strong positivity of PTEN around follicles (arrows) with weak expression in lymphoma cells within the follicles (stars), reactive T cells are positive (arrowheads) (magnification is × 40 and × 630 for the left and right panels, respectively).

The expression of pAKT and PTEN was examined using immunohistochemistry in the corresponding paraffin-embedded FL samples. Positive pAKT immunostaining of variable intensity was observed in intra-follicular malignant B-cells from 15/38 FL samples (Figure 3A). Among these 15 cases, rare interfollicular reactive T-cells showed positive pAKT staining in one case. 18/38 FL specimens were negative for pAKT staining, whereas the IHC status could not be assessed in 5 specimens.PTEN immunostaining was positive in malignant intra-follicular B-cells in 30/38 cases, and 18 out of these 30 samples also displayed the same intensity of PTEN positivity in reactive interfollicular T-cells and macrophages. PTEN signals were heterogeneous in 12 out of these 30 samples, displaying either strong PTEN positivity in malignant B-cell follicles associated with low staining of reactive T-cells and macrophages around the follicles, or an inverse profile of positivity (Figure 3B and C). PTEN staining was negative in 3/38 specimens, whereas the IHC status could not be assessed in 5 specimens.

The benign lymphadenitis cases showed homogeneous positive staining of PTEN. For the pAKT staining, case #39 had rare positive cells, and case #40 showed negative staining of pAKT.

#### Correlations between IHC and WB

The 8 PTEN WB-negative cases showed different pattern of IHC expression: 1 case was completely negative; one case was not evaluable due to high background, whereas the other cases showed heterogeneous expression in a fraction of malignant or reactive cells.

Although IHC data correlated with WB results in most samples, it is noteworthy that the correlation was not perfect. This may be due to the different levels of sensitivity of each method and/or to the intra-tumoral heterogeneity. In this extent, WB appeared more reliable to evaluate the level of pAKT and PTEN expression because it reflects an overall average amount of each protein, which could be quantified using densitometry. In contrast, it was not possible to assess a reliable positive cut-off for IHC expression due to the variations in the number of positive cells within sections. However, IHC was useful and necessary to localize the expression in malignant and /or reactive cells. The results are summarized in Table 2.

**Table 2:** **Summary of western blot analysis of AKT, pAKT, and PTEN expression**

Samples	AKT expression	pAKT expression	PTEN expression
**1**	**+**	**-**	**+**
**2**	**+**	**-**	**+**
**3**	**+**	**-**	**+**
**4**	**+**	**+**	**+**
**5**	**+**	**-**	**+**
**6**	**+**	**+**	**+**
**7**	**+**	**+**	**+**
**8**	**+**	**+**	**+**
**9**	**+**	**+**	**+**
**10**	**+**	**-**	**-**
**11**	**+**	**-**	**+**
**12**	**+**	**+**	**+**
**13**	**+**	**+**	**+**
**14**	**+**	**+**	**+**
**15**	**+**	**-**	**+**
**16**	**+**	**-**	**+**
**17**	**+**	**-**	**-**
**18**	**+**	**-**	**+**
**19**	**+**	**-**	**+**
**20**	**+**	**+**	**+**
**21**	**+**	**-**	**+**
**22**	**+**	**-**	**+**
**23**	**+**	**-**	**+**
**24**	**+**	**-**	**+**
**25**	**+**	**-**	**+**
**26**	**+**	**-**	**-**
**27**	**+**	**-**	**+**
**28**	**+**	**-**	**+**
**29**	**+**	**+**	**+**
**30**	**+**	**+**	**-**
**31**	**+**	**+**	**-**
**32**	**+**	**+**	**-**
**33**	**+**	**-**	**-**
**34**	**+**	**-**	**+**
**35**	**+**	**-**	**+**
**36**	**+**	**-**	**-**
**37**	**+**	**-**	**+**
**38**	**+**	**+**	**+**
**39**	**+**	**+**	**+**
**40**	**+**	**+**	**+**

Follicular Lymphoma samples (#1-38); lymphadenitis samples (#39 and 40).All samples are wild type for the mutation of PIK3CA gene. **+**: expression; **-**: no expression.

### Correlation with clinical features

We were interested in testing the relationship between pAKT expression status and clinicopathological parameters like histological grade, tumor volume, overall survival (OS), and progression free survival (PFS). No significant correlations were found with any parameters. The clinical features of patients are summarized in Table 1.

## Discussion

The PI3K/AKT pathway is frequently activated in human carcinomas by oncogenic mutations of the PIK3CA gene encoding for the α isoform of the PI3K p110 subunit (24). Only a few studies have focused on the PI3K/AKT pathway in lymphoid neoplasms. It was reported to be activated in DLBCL [25, 26] and MCL [27, 28] due to the presence of phosphorylated AKT (Ser473). As to FL, an increase of phosphorylated AKT (Ser473) has also been shown in two different reports using a proteomic approach by laser capture microdissection and reverse phase protein microarray, but the mutational status of PI3K was not addressed [29, 30]. Due to the scarcity of data in the literature, we aimed in the present study to further clarify the abnormalities of the PI3K/AKT pathway in FL.

We could detect phosphorylation of AKT using WB in 37% of FL samples, most of them displaying high level of pAKT expression. This finding is in accordance with those previously obtained using a reverse phase protein microarray approach [29, 30], suggesting that activation of the PI3K/AKT pathway might contribute in the development of FL.

Of note, correlations between WB and IHC data were not strictly correlated, probably due to the visual scoring used for IHC analysis, which resulted in less accurate quantification than the automatic densitometry method used for WB analysis. This may be also explained by the requirement of different antibodies and by the different threshold levels of sensitivity in each method. WB results were considered for subsequent statistical and clinical correlations because the intra-tumoral heterogeneity can account for a biased pattern of IHC expression in a single tissue section, whereas WB is more likely to accurately reflect a given level of protein expression in the whole tissue sample. IHC was however useful to confirm that pAKT and PTEN expression were located in malignant and/or reactive cells. We did not find any correlations of pAKT WB expression with clinicopathological data, but this could be attributed to the relatively small number of cases in our series.

Aberrant regulation of the PI3K/AKT signaling pathway in MCL occurs most frequently by loss of PTEN expression and/or PIK3CA gene amplification, whereas the PIK3CA gene was found unmutated [27, 28]. Similarly, no activating mutations of PIK3CA gene have been identified in our series of FL. Since we analyzed only hot spot loci in both the helical and kinase domains, we cannot rule out the hypothesis that some mutations could be located in other domains. This seems unlikely since the hot spot loci are common to a wide variety of human tumors [10, 11, 23, 36].

Altogether, these results suggest that PIK3CA mutations are not a frequent AKT activating mechanism in lymphoid neoplasm. We have thus searched for another mechanism of AKT activation. In our cohort, we found loss of PTEN in 3 cases with activated AKT, including 2 cases with a high level of pAKT expression. However only a minority of pAKT activated FL samples displayed loss of PTEN, which argues against a crucial role of PTEN dysfunction in FL pathogenesis. Activation of PI3K/AKT pathway may thus occur in FL through alternative events previously evidenced in other cancer types, including mutation or gene amplification of membrane receptors, overexpression of growth factors, AKT gene amplification, and PIK3CA gene amplification [37, 38, 18]. The latter abnormality has been demonstrated in MCL as an additional mechanism of AKT activation [28]. As to FL, AKT activation may also be related to PI3Kδ. Although no mutation of PI3Kδ was previously found [39], it is expressed at high levels in lymphocytes and lymphoid tissue [40], and is involved in the survival of B cells [41]. It will be interesting to further analyze phosphorylation events in FL patient samples at the single cell level using detection of phospho-epitopes like phosphoflow analysis [42].

Of note, loss of PTEN expression was also observed in 5 samples lacking pAKT expression. This suggests that loss of PTEN expression may arise in FL without subsequent AKT activation. This may be related to negative regulation of AKT by direct dephosphorylation of the hydrophobic motif by the phosphatase PHLPP [43].

## Conclusions

The present report suggests that the PI3K/AKT pathway may be constitutively activated in some FL cases in the absence of PIK3CA mutation. However, since these abnormalities were detected in only a fraction of FL cases, they may be not sufficient to explain previous observations that a dual PI3K and mTOR inhibitor (NVP-BEZ235) has a potential efficiency against FL cell lines [2] and that the PI3Kδ inhibitor idelalisib (CAL- 101) has clinical activity against FL [31–33]. Further studies are thus needed to better understand the mechanisms involved by drugs targeting AKT and PI3K in FL.

## Abbreviations

DLBCL: Diffuse large B cell lymphoma
FL: Follicular lymphoma
IHC: Immunohistochemistry
MCL: Mantle cell lymphoma
NHL: Non Hodgkin lymphoma
OS: Overall survival
pAKT: Phosphorylated AKT
PCR: Polymerase Chain Reaction
PFS: Progression free survival
PI3K: Phosphoinositide 3- kinase
PIP3: Phosphatidylinositol-3, 4, 5-trisphosphate
PTEN: Phosphatase and tensin homolog
WB: Western blot.
